# India-Rubber in Brazil

**Published:** 1884-08

**Authors:** 


### INDIA RUBBER IN BRAZIL.

IN the early morning men and women come with baskets of clay cups on their backs, and little hatchets to gash the trees. Where the white milk drips down from the gash they stick their cups on the trunk with daubs of clay, moulded so as to catch the whole flow. If the tree is a large one four or five gashes may be cut in a circle around the trunk. On the next day other gashes are made a little below these, and so on until the rows reach the ground. By eleven o'clock the flow of milk has ceased, and the "seringueiros" come to collect the contents of the cups in calabash jugs. A gill or so is the utmost yield from each tree, and a single gatherer may attend to a hundred and twenty trees or more, wading always through these dark marshes, and paying dearly for his profit in fever and weakness. Our "mamelnca" hostess has brought in her day's gathering—a calabash full of the white liquid, in appearance precisely like milk. If left in this condition it coagulates after a while and forms an inferior whitish gum. To make the black rubber of commerce the milk must go through a peculiar process of manufacture, for which our guide has been preparing. Over a smouldering fire, fed with hard nuts of the "tecuma" palm, he places a kind of a clay chimney, like a wide-mouthed bottomless jug; through this "boiao" the thick smoke pours in a constant stream. Now he takes his mould—in this case wooden, like a round-bladed paddle—washes it in the milk, and holds it over the smoke until the liquid coagulates. Then another coat is added, only now, as the wood is heated, the milk coagulates faster. It may take the gatherings of two or three days to cover the mould thickly enough. Then the rubber is still dull white, but in a short time it turns brown, and finally almost black, as it is sent to the market.

The mass is cut from the paddle and sold to traders in the village. Bottles are sometimes made by moulding the rubber over a clay ball, which is then broken up and removed. Twenty million pounds of rubber, valued at $6,000,000, are annually exported from Para in the dry season; many thousand people are engaged in gathering it. But the business altogether is a ruinous one for the province, as Brazilians themselves are fully aware. The "seringueiro," who gains two or three dollars for a single day's gathering, has enough, as life goes here, to keep him in idleness for a week; and when his money is spent he can draw again on his ever-ready bank.

The present wasteful system is spoken of as follows: The half wild "seringueiros" will go on submitting to impositions and dying here in the swamps, until Brazilians learn that by purchasing this land from the government and planting it in rubber trees they can insure vastly greater profits, and do away with the evils of the present system. It is what must eventually be done. The rubber gatherers, in their eagerness to secure large harvests, have already killed an immense number of trees about the Para estuary ; they have been obliged to penetrate farther and farther into the forest, to the Tocantins, Madeira, Purus, Rio Negro, and eventually even these regions must be exhausted unless they are protected in some way. The trees, properly planted and cared for, will yield well in fifteen years, and of course the cost of gathering would be vastly reduced in a compact plantation. Half the present labor of the rubber collector consists in his long tramps through the swampy forest.—[*Dominica Dial.*

